# Supplementary material for: Perturbing local steroidogenesis to improve breast cancer immunity
Source: Nat Commun. 2025 Apr 26;16:3945. doi: 10.1038/s41467-025-59356-3 (PMC12033260; doi:10.1038/s41467-025-59356-3)
Supplement: Supplementary file 1 — Supplementary Information [file 41467_2025_59356_MOESM1_ESM.pdf]

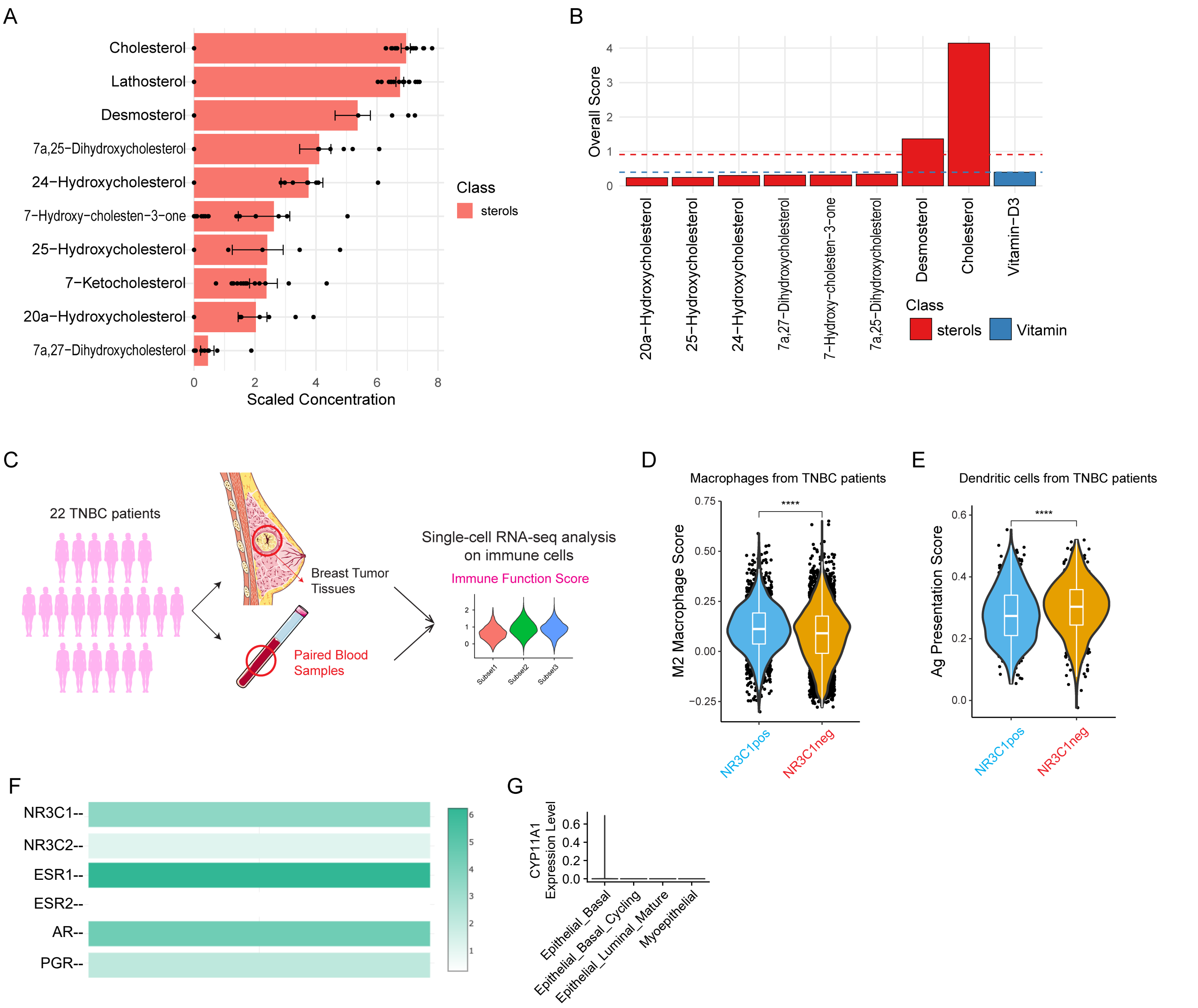
A
Cholesterol
Lathosterol
Desmosterol
7a,25−Dihydroxycholesterol
24−Hydroxycholesterol
7−Hydroxy−cholesten−3−one
25−Hydroxycholesterol
7−Ketocholesterol
20a−Hydroxycholesterol
7a,27−Dihydroxycholesterol
0 2 4 6 8
Scaled Concentration
Class
sterols
B
Overall Score
20a−Hydroxycholesterol
25−Hydroxycholesterol
24−Hydroxycholesterol
7a,27−Dihydroxycholesterol
7−Hydroxy−cholesten−3−one
7a,25−Dihydroxycholesterol
Desmosterol
Cholesterol
Vitamin−D3
Class
sterols
Vitamin
C
22 TNBC patients
Breast Tumor Tissues
Paired Blood Samples
Single-cell RNA-seq analysis on immune cells
Immune Function Score
D
Macrophages from TNBC patients
M2 Macrophage Score
NR3C1pos
NR3C1neg
E
Dendritic cells from TNBC patients
Ag Presentation Score
NR3C1pos
NR3C1neg
F
NR3C1--
NR3C2--
ESR1--
ESR2--
AR--
PGR--
G
CYP11A1 Expression Level
Epithelial_Basal
Epithelial_Basal_Cycling
Epithelial_Luminal_Mature
Myoepithelial

Supplementary Figure 1. Local Steroidogenesis and Steroid Signalling in TNBC Tumours.

A. Bar chart highlighting the scaled concentrations of 9 distinct sterols detected in the 16 TNBC tumours via LC-MS after organic solvent extraction.

B. Bar chart indicating the overall steroid hormone score derived by integrating steroid hormone concentrations and gene expression data in TNBC tumours.

C. A schematic diagram depicting the scRNA-seq data analysis from TNBC patients.

D-E. Violin plot detailing the comparative levels of indicated signature scores in *NR3C1*+/- immune cells derived from tumour samples, based on data from 22 TNBC patients.

F. Bar plot illustrating the expression levels of various steroid hormone receptors in tumours from patients with breast invasive carcinoma, analysed using the GEPIA tool to access TCGA datasets.

G. Violin plot depicting the expression level of CYP11A1 within epithelial cells from 5 TNBC patients.

Note: Statistical significance indicators: $*p < 0.05$, $**p < 0.01$, $***p < 0.001$, $****p < 0.0001$.

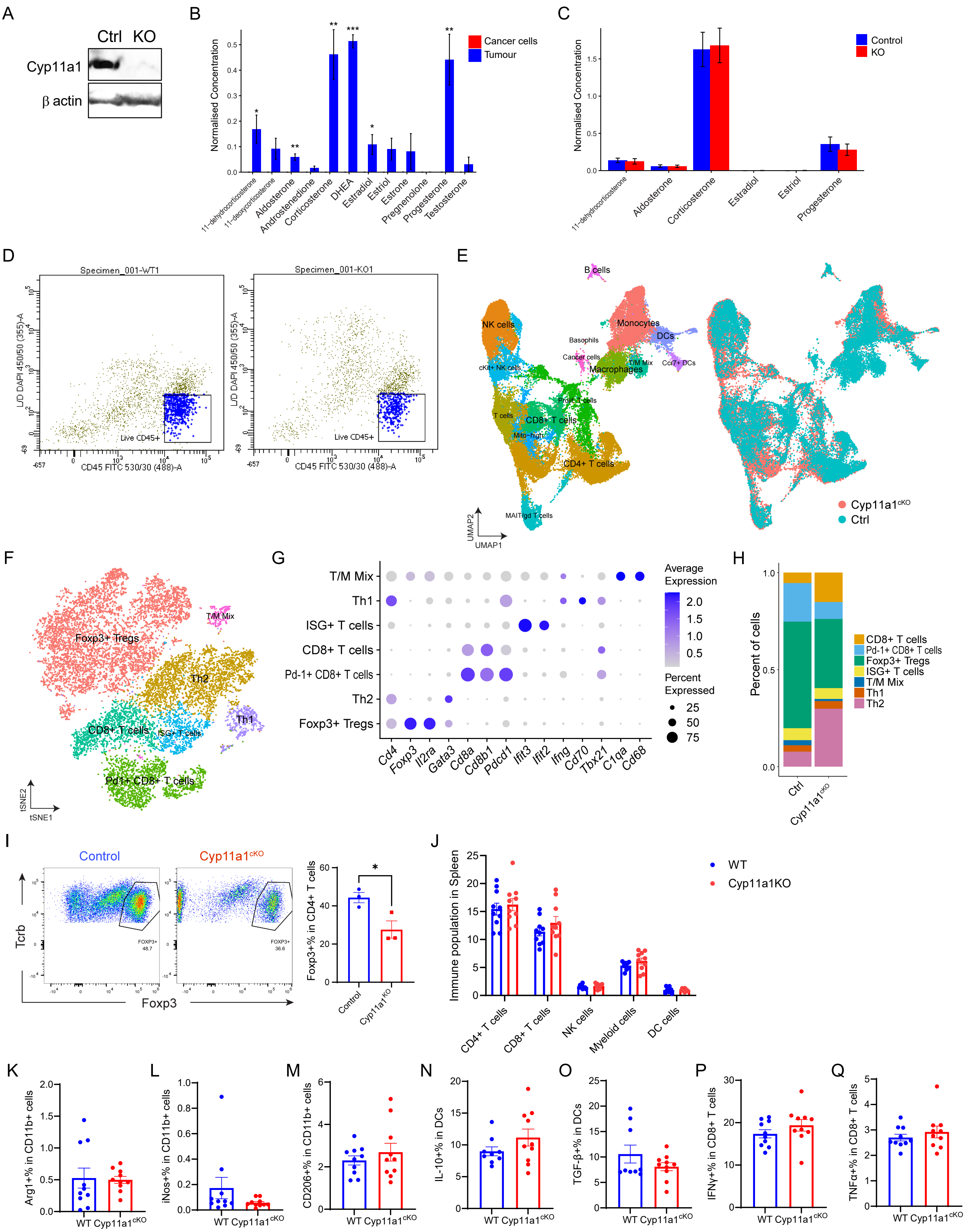
A
Ctrl KO
Cyp11a1
β actin
B
Cancer cells
Tumour
Normalised Concentration
11-dehydrocorticosterone
11-deoxycorticosterone
Aldosterone
Androstenedione
Corticosterone
DHEA
Estradiol
Estriol
Estrone
Pregnenolone
Progesterone
Testosterone
C
Control
KO
D
Specimen_001-WT1
Specimen_001-KO1
L/D DAPI 450/50 (355)-A
CD45 FITC 530/30 (488)-A
Live CD45+
E
B cells
NK cells
Monocytes
DCs
Basophils
Cancer cells
cKit+ NK cells
Macrophages
T/M Mix
Ccr7+ DCs
T cells
CD8+ T cells
Mito-high
CD4+ T cells
MAIT/gd T cells
UMAP2
UMAP1
Cyp11a1cKO
Ctrl
F
T/M Mix
Foxp3+ Tregs
Th2
Th1
CD8+ T cells
ISG+ T cells
Pd1+ CD8+ T cells
tSNE2
tSNE1
G
T/M Mix
Th1
ISG+ T cells
CD8+ T cells
Pd-1+ CD8+ T cells
Th2
Foxp3+ Tregs
Cd4
Foxp3
Il2ra
Gata3
Cd8a
Cd8b1
Pdcd1
Ifit3
Ifit2
Ifng
Cd70
Tbx21
C1qa
Cd68
Average Expression
Percent Expressed
H
Percent of cells
CD8+ T cells
Pd-1+ CD8+ T cells
Foxp3+ Tregs
ISG+ T cells
T/M Mix
Th1
Th2
Ctrl
Cyp11a1cKO
I
Control
Cyp11a1cKO
Tcrb
Foxp3
FOXP3+ 48.7
FOXP3+ 36.6
Foxp3+% in CD4+ T cells
Control
Cyp11a1KO
J
Immune population in Spleen
WT
Cyp11a1KO
CD4+ T cells
CD8+ T cells
NK cells
Myeloid cells
DC cells
K
Arg1+% in CD11b+ cells
L
iNos+% in CD11b+ cells
M
CD206+% in CD11b+ cells
N
IL-10+% in DCs
O
TGF-β+% in DCs
P
IFNγ+% in CD8+ T cells
Q
TNFα+% in CD8+ T cells
WT Cyp11a1cKO

Supplementary Figure 2. Genetic Deletion of Cyp11a1 in Immune Cells Alters TNBC Tumour Dynamics and the Immune Landscape of the TME.

A. Western blot analysis of Cyp11a1 expression in splenocytes from control and Cyp11a1^{cKO} mice. β-actin used as a loading control.

B. Bar chart showing the scaled concentrations of steroid hormones detected in the E0771.LMB cells and tumours from control mice via LC-MS after organic solvent extraction (N=3 for cancer cells and N=6 for tumour tissues).

C. Bar chart showing the scaled concentrations of steroid hormones detected in the serum samples from control and Cyp11a1^{cKO} mice via LC-MS after organic solvent extraction (N=10).

D. Flow cytometry visualizations indicating cell sorting strategy for scRNA-seq.

E. UMAP plots of CD45^{+} immune cells from control (N=3) and Cyp11a1^{cKO} (N=3) mice. Different colours on the left highlight distinct immune cell subsets, while the right contrasts cell origins from control versus Cyp11a1^{cKO} mice.

F. t-SNE plots of T cells from control (N=3) and Cyp11a1^{cKO} (N=3) mice. Different colours highlight distinct T cell subsets.

G. Dot plot visualization capturing marker gene expression variations across diverse T cell clusters.

H. Bar plot showing the relative contributions of T cell clusters from control and Cyp11a1^{cKO} mice.

I. Flow cytometry visualizations indicating Foxp3 expression in CD4^{+} cells of Vav1^{Cre} versus Cyp11a1^{cKO} mice (left). The subsequent chart (right) illustrates the comparative percentage expression in both mouse types (N=3).

J. Comparative analysis of immune cell populations in the spleen samples from control and Cyp11a1^{cKO} mice. The graph shows the percentage of CD4^{+} T cells, CD8^{+} T cells, NK cells, myeloid cells, and dendritic cells in the spleen.

K-Q. Bar illustrates the comparative percentage expression of Arg1, iNos, and CD206 in CD11b+ cells (K-M), IL-10 and TGF-β in DCs (N-O), and IFN-γ and TNF-α in CD8^{+} T cells from the spleen samples from control and Cyp11a1^{cKO} mice.

Note: Data are presented as mean ± SEM; Significance indicators utilized are: *p < 0.05, **p < 0.01, ***p < 0.001, and ****p < 0.0001. Source data for A, B, C, I, J are provided as a Source Data file.

Supplementary Figure 3

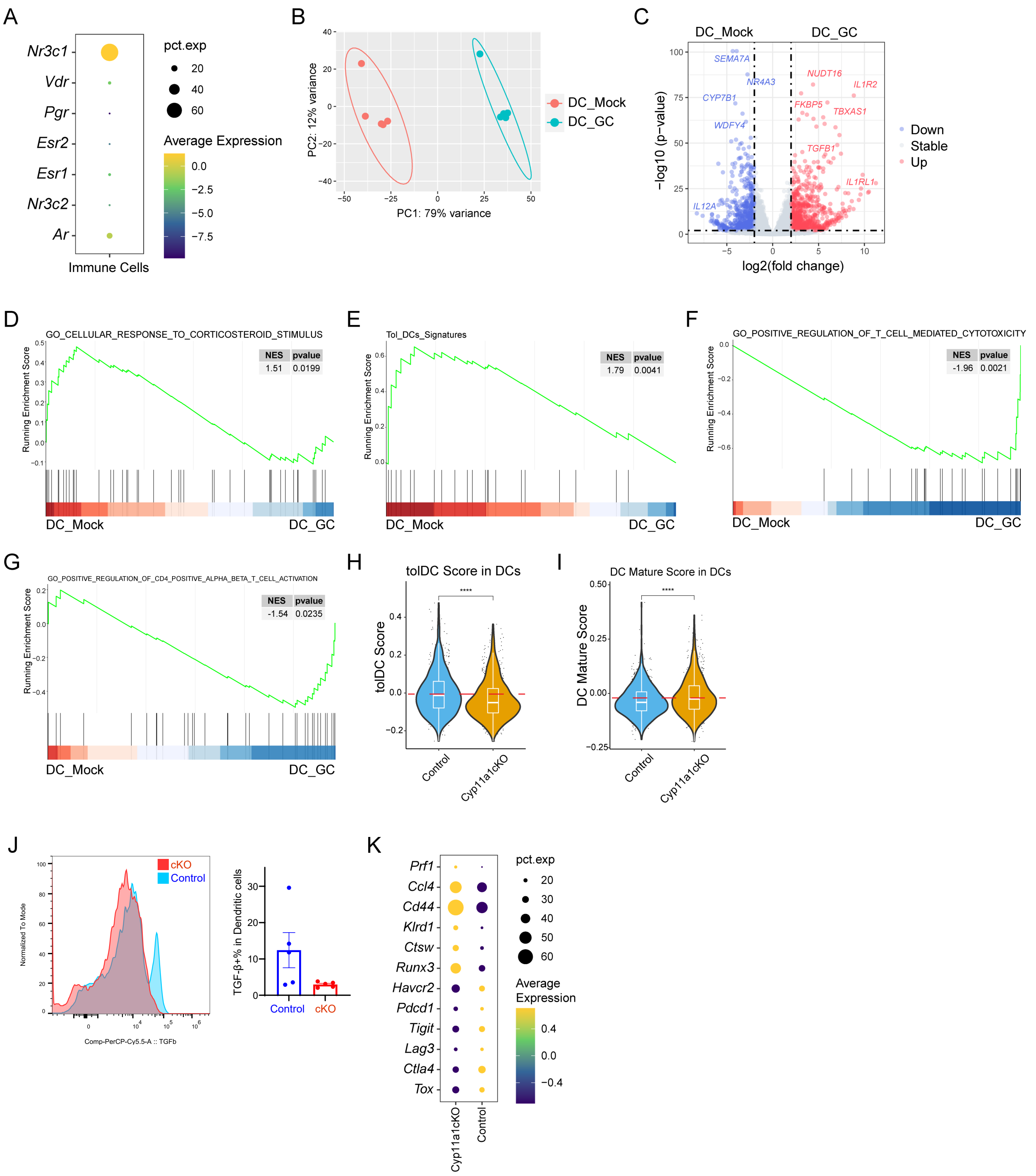

Supplementary Figure 3: Steroidogenesis Inhibition Augments Anti-Tumour Immunity by Suppressing Glucocorticoid Signalling

A. Dot plots delineate the prevalence of diverse steroid hormone receptor genes in tumour-infiltrating immune cells from TNBC mouse model.

B. Feature plots provide a visual comparison of *Tsc22d3* expression within immune cells of $Vav1^{Cre}$ and $Cyp11a1^{cKO}$ mice.

C. A volcano plot depicting the gene expression modifications in DCs post-GC treatment. D-G. GSEA results of indicated gene sets in DCs from mock and GC-treated groups.

H-I. Violin plots reveal the relative significance of indicated gene set signature scores in DCs and T cells, drawn from either $Vav1^{Cre}$ or $Cyp11a1^{cKO}$ mice.

J. FACS histogram on the left, displaying the expression intensity of TGF-β in DCs from $Vav1^{Cre}$ or $Cyp11a1^{cKO}$ mice. The complementary right panel juxtaposes percentage expression in both cohorts (N = 5; Mean ± SEM demarcated by error bars); Source data are provided as a Source Data file.

K. Dot plot presentation of indicated gene expression levels in $CD8^{+}$ T cells sourced from both $Vav1^{Cre}$ or $Cyp11a1^{cKO}$ mice.

Notes: Significance levels denoted as: $*p < 0.05$, $**p < 0.01$, $***p < 0.001$, $****p < 0.0001$.

Supplementary Figure 4

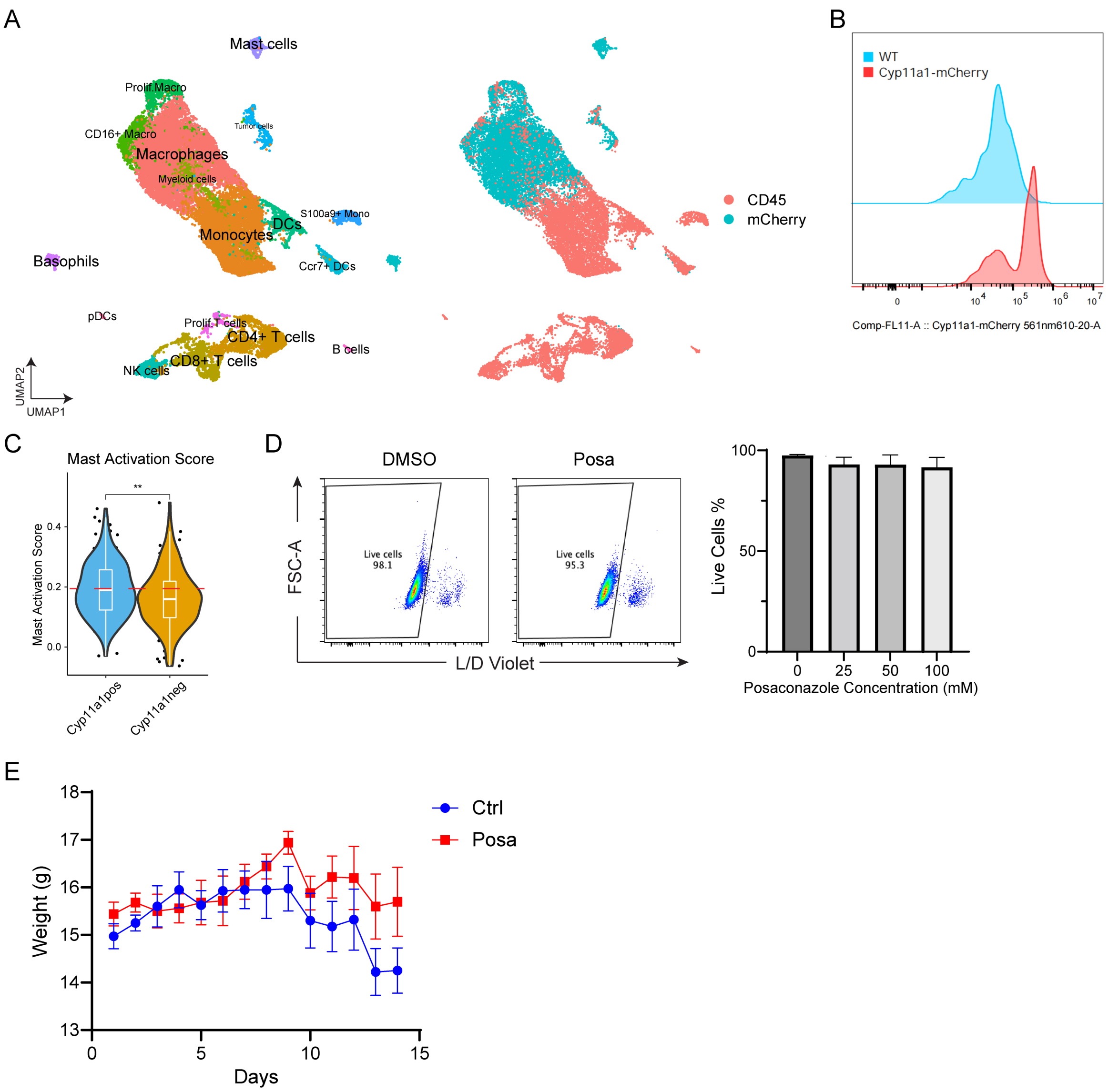

Supplementary Figure 4: Characterization of Cyp11a1^{+} immunocytes in TNBC for enhanced therapeutic precision

A. UMAP visualization of distinct clusters in CD45^{+}mCherry^{+} and CD45^{+}mCherry^{-} immune cells from the reporter mice. Different colours on the left highlight distinct immune cell subsets, while the right contrasts cell origins from CD45^{+}mCherry^{+} and mCherry^{-}population.

B. Representative FACS profile to show mCherry expression in tumour-infiltrating FceR1^{+} cKit^{+} SiglecF^{-} mast cells in E0771.LMB syngeneic tumour bearing mice.

C. Violin plot detailing the comparative levels of mast cell activation signature score in *Cyp11a1*$^{+/-}$ mast cells derived from tumour samples.

D. Flow cytometry graphs display FSC-A against L/D Violet staining, comparing DMSO and Posaconazole-treated mast cells. The adjacent chart quantifies the percentage of live cells across varying concentrations (N = 4).

E. Longitudinal weight curves in Posaconazole-treated and control mice over time (N = 9). Source data are provided as a Source Data file.

Notes: Significance levels denoted as: $*p < 0.05$, $**p < 0.01$, $***p < 0.001$, $****p < 0.0001$.

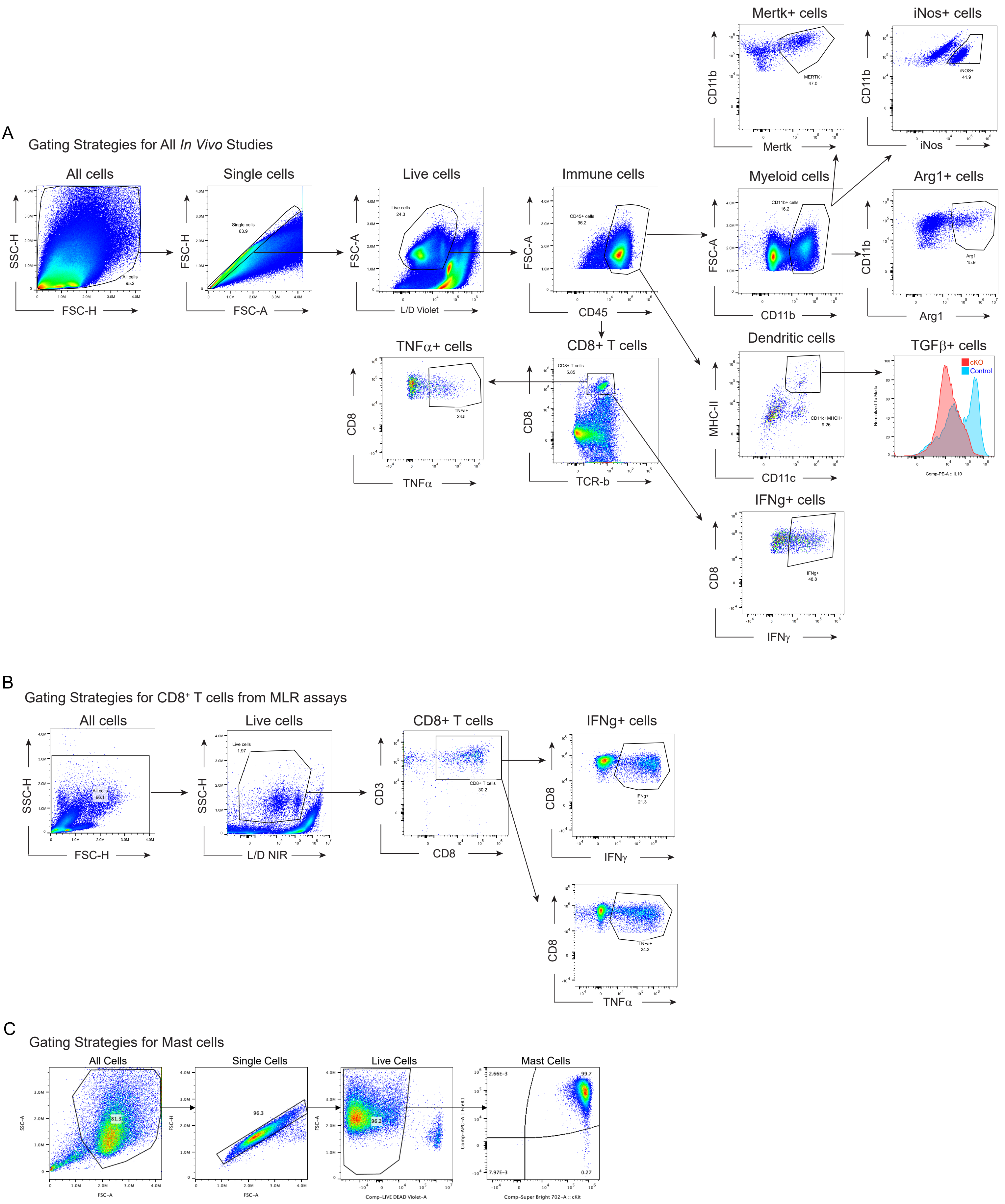

Figure S5. Gating Strategies for All Flow Cytometry Experiments in This Study.

A. Gating strategy for all *in vivo* studies.

B. Gating strategy for $CD8^+$ T cells from MLR assays.

C. Gating strategy for mast cells purity check.
