# Supplementary material for: Perturbing local steroidogenesis to improve breast cancer immunity
Source: Nat Commun. 2025 Apr 26;16:3945. doi: 10.1038/s41467-025-59356-3 (PMC12033260; doi:10.1038/s41467-025-59356-3)
Supplement: Supplementary file 4 — Reporting Summary [file 41467_2025_59356_MOESM4_ESM.pdf]

nature portfolio

Corresponding author(s): NCOMMS-24-09549-T

Last updated by author(s): Feb 23, 2024

Reporting Summary

Nature Portfolio wishes to improve the reproducibility of the work that we publish. This form provides structure for consistency and transparency in reporting. For further information on Nature Portfolio policies, see our Editorial Policies and the Editorial Policy Checklist.

Statistics

For all statistical analyses, confirm that the following items are present in the figure legend, table legend, main text, or Methods section.

n/a	Confirmed	
☐	☒	The exact sample size (*n*) for each experimental group/condition, given as a discrete number and unit of measurement
☐	☒	A statement on whether measurements were taken from distinct samples or whether the same sample was measured repeatedly
☐	☒	The statistical test(s) used AND whether they are one- or two-sided<br>*Only common tests should be described solely by name; describe more complex techniques in the Methods section.*
☒	☐	A description of all covariates tested
☐	☒	A description of any assumptions or corrections, such as tests of normality and adjustment for multiple comparisons
☐	☒	A full description of the statistical parameters including central tendency (e.g. means) or other basic estimates (e.g. regression coefficient) AND variation (e.g. standard deviation) or associated estimates of uncertainty (e.g. confidence intervals)
☐	☒	For null hypothesis testing, the test statistic (e.g. F, t, r) with confidence intervals, effect sizes, degrees of freedom and P value noted<br>*Give P values as exact values whenever suitable.*
☒	☐	For Bayesian analysis, information on the choice of priors and Markov chain Monte Carlo settings
☒	☐	For hierarchical and complex designs, identification of the appropriate level for tests and full reporting of outcomes
☐	☒	Estimates of effect sizes (e.g. Cohen's d, Pearson's r), indicating how they were calculated

Our web collection on statistics for biologists contains articles on many of the points above.

Software and code

Policy information about availability of computer code

Data collection	Cytek Aurora (5L) flow cytometer, MoFlo XDP sorter, BD Influx cytometer, Illumina NovaSeq6000.
Data analysis	FlowJo v10.2 software, Graph Pad Prism 9, Cell Ranger toolkit 7.1.0, Seurat 3.2.2, Compass, HISAT2 aligner 2.2.1, Samtools 1.9, DESeq2 1.30.1, clusterProfiler 3.18.1, ggpubr 0.4.0.

For manuscripts utilizing custom algorithms or software that are central to the research but not yet described in published literature, software must be made available to editors and reviewers. We strongly encourage code deposition in a community repository (e.g. GitHub). See the Nature Portfolio guidelines for submitting code & software for further information.

Data

Policy information about availability of data

All manuscripts must include a data availability statement. This statement should provide the following information, where applicable:

- Accession codes, unique identifiers, or web links for publicly available datasets
- A description of any restrictions on data availability
- For clinical datasets or third party data, please ensure that the statement adheres to our policy

Processed single cell RNA-seq and bulk RNA-seq data of this study can be obtained from Gene Expression Omnibus (GEO) with an accession number of GSE255495. The scRNA-seq data derived from both tumour tissues and matched blood samples of TNBC patients utilised across all studies are publicly accessible via the GEO under the accession number GSE169246. The RNA-seq data from tumours of TNBC patients is accessible via Sequence Read Archive (SRA) under the accession number SRP157974.

Research involving human participants, their data, or biological material

Policy information about studies with human participants or human data. See also policy information about sex, gender (identity/presentation), and sexual orientation and race, ethnicity and racism.

Reporting on sex and gender	Only female TNBC patients were included.
Reporting on race, ethnicity, or other socially relevant groupings	Chinese ethnicity.
Population characteristics	*Describe the covariate-relevant population characteristics of the human research participants (e.g. age, genotypic information, past and current diagnosis and treatment categories). If you filled out the behavioural & social sciences study design questions and have nothing to add here, write "See above."*
Recruitment	Eligibility criteria included a histological confirmation of TNBC, no prior exposure to anti-tumour therapies, and surgical treatment.
Ethics oversight	All research protocols were approved by the Ethical Committee of Shandong Cancer Hospital and Institute, affiliated with Shandong First Medical University and Shandong Academy of Medical Sciences (Approval No. SDTHEC2021003035). Every participant provided written informed consent, permitting tissue collection and subsequent metabolomics profiling.

Note that full information on the approval of the study protocol must also be provided in the manuscript.

Field-specific reporting

Please select the one below that is the best fit for your research. If you are not sure, read the appropriate sections before making your selection.

☒ Life sciences ☐ Behavioural & social sciences ☐ Ecological, evolutionary & environmental sciences

For a reference copy of the document with all sections, see nature.com/documents/nr-reporting-summary-flat.pdf

Life sciences study design

All studies must disclose on these points even when the disclosure is negative.

Sample size	The sample size was determined according to our previous experience and a priori power analysis (G*Power).
Data exclusions	*Describe any data exclusions. If no data were excluded from the analyses, state so OR if data were excluded, describe the exclusions and the rationale behind them, indicating whether exclusion criteria were pre-established.*
Replication	Experiments were repeated and detailed in the Figure legends.
Randomization	Genetically altered mice were sorted according to their genotype for comparison with control littermates. Whenever feasible, mice were matched for age and sex.
Blinding	Researchers and animal technicians were kept blinded throughout the experiments whenever feasible. The association between animal IDs and genotypes was established at the conclusion of each experiment.

Reporting for specific materials, systems and methods

We require information from authors about some types of materials, experimental systems and methods used in many studies. Here, indicate whether each material, system or method listed is relevant to your study. If you are not sure if a list item applies to your research, read the appropriate section before selecting a response.

Materials & experimental systems

n/a	Involved in the study	
☐	☒	Antibodies
☐	☒	Eukaryotic cell lines
☒	☐	Palaeontology and archaeology
☐	☒	Animals and other organisms
☐	☒	Clinical data
☒	☐	Dual use research of concern
☒	☐	Plants

Methods

n/a	Involved in the study	
☒	☐	ChIP-seq
☐	☒	Flow cytometry
☒	☐	MRI-based neuroimaging

Antibodies

Antibodies used	Human CD86 (PE), IFNγ (AF-700), and TNFα (PE-Cy7); Mouse CD45 (BV711, BUV563 & FITC), CD11b (APC-Cy7), Mertk (PE-Cy7), iNOS (APC), TCRb (eFluor 450 & FITC), CD4 (APC & PE-Cy7), Foxp3 (PE), CD8 (APC-eFluor 780), IFNg (PerCp-Cy5.5), TNFa (PE-eFluor 610), FceR1 (FITC), cKit (BV711), SiglecF (PerCP-eFluor 710), along with Live/Dead Fixable Violet (Thermo).
Validation	Antibody validation information for each of the listed antibodies can be found on the respective manufacturer's website.

Eukaryotic cell lines

Policy information about cell lines and Sex and Gender in Research

Cell line source(s)	The E0771.LMB cell line was generously provided by Robin Anderson. Human triple-negative breast cancer cells MDA-MB-231-Luc were purchased from Anwei-sci Cell Center, Shanghai, China. (Item No. AW-CELLS-H0219).
Authentication	STR tested.
Mycoplasma contamination	All cell lines were screened and negative for mycoplasma.
Commonly misidentified lines (See ICLAC register)	No commonly misidentified cell lines were used in the study.

Animals and other research organisms

Policy information about studies involving animals; ARRIVE guidelines recommended for reporting animal research, and Sex and Gender in Research

Laboratory animals	Wild type: C57BL/6 aged between 8 to 12 weeks. Genetically modified: Vav-1-Cre;Cyp11a1fl/fl and Cyp11a1-mCherry reporter mice aged between 8 to 12 weeks; NOG-dKO (murine MHC class I- and class II-deficient) mice aged 6 weeks.
Wild animals	No wild animals were used in the study.
Reporting on sex	Only female mice were used.
Field-collected samples	No field collected samples were used in the study.
Ethics oversight	In this investigation, normal mice were handled and cared for following the stringent guidelines set forth by the UK Animals in Science Regulation Unit's Code of Practice for the Housing and Care of Animals Bred, Supplied, or Used for Scientific Purposes, and the Animals (Scientific Procedures) Act 1986 Amendment Regulations 2012. All experimental protocols were conducted under the authorization of a UK Home Office Project License (PPL P0AB4361E) and received the necessary approval from the local institute's Animal Welfare and Ethical Review Body, ensuring compliance with ethical standards and animal welfare considerations.

Note that full information on the approval of the study protocol must also be provided in the manuscript.

Clinical data

Policy information about clinical studies

All manuscripts should comply with the ICMJEguidelines for publication of clinical research and a completedCONSORT checklist must be included with all submissions.

Clinical trial registration	*Provide the trial registration number from ClinicalTrials.gov or an equivalent agency.*
Study protocol	*Note where the full trial protocol can be accessed OR if not available, explain why.*
Data collection	*Describe the settings and locales of data collection, noting the time periods of recruitment and data collection.*
Outcomes	*Describe how you pre-defined primary and secondary outcome measures and how you assessed these measures.*

Plants

Seed stocks	*Report on the source of all seed stocks or other plant material used. If applicable, state the seed stock centre and catalogue number. If plant specimens were collected from the field, describe the collection location, date and sampling procedures.*
Novel plant genotypes	*Describe the methods by which all novel plant genotypes were produced. This includes those generated by transgenic approaches, gene editing, chemical/radiation-based mutagenesis and hybridization. For transgenic lines, describe the transformation method, the number of independent lines analyzed and the generation upon which experiments were performed. For gene-edited lines, describe the editor used, the endogenous sequence targeted for editing, the targeting guide RNA sequence (if applicable) and how the editor was applied.*
Authentication	*Describe any authentication procedures for each seed stock used or novel genotype generated. Describe any experiments used to assess the effect of a mutation and, where applicable, how potential secondary effects (e.g. second site T-DNA insertions, mosiacism, off-target gene editing) were examined.*

Flow Cytometry

Plots

Confirm that:

- [x] The axis labels state the marker and fluorochrome used (e.g. CD4-FITC).
- [x] The axis scales are clearly visible. Include numbers along axes only for bottom left plot of group (a 'group' is an analysis of identical markers).
- [x] All plots are contour plots with outliers or pseudocolor plots.
- [x] A numerical value for number of cells or percentage (with statistics) is provided.

Methodology

Sample preparation	Cells prepared for flow cytometric analysis were subjected to a 4-hour incubation with PMA (50 ng/ml) and Ionomycin (500 ng/ml), followed by the addition of Monensin (Biolegend) to the culture for the last 3 hours. The established protocols for surface staining and intracellular cytoplasmic protein staining by eBioscience were diligently followed. LIVE/DEAD Fixable Dead Cell Stain (Thermo Fisher) was employed to stain the single-cell suspensions, which were subsequently fixed using the eBioscience IC fixation buffer. For intracellular cytokine staining, the cells were fixed and permeabilized using eBioscience IC Fixation buffer and 1x permeabilization buffer, respectively. Fluorescent dye-conjugated antibodies were then applied to stain the cells. After a thorough wash with 3% PBS-FCS, the cells were ready for analysis using the Cytek Aurora (5L) flow cytometer. FlowJo v10.2 software facilitated the data analysis.
Instrument	Cytek Aurora (5L) flow cytometer
Software	FlowJo v10.2 software
Cell population abundance	Purity levels exceeding 98% are routinely achieved and determined through flow cytometry analysis of post-sorted cells.
Gating strategy	The cells were initially gated based on the cell population in the FSC/SSC plot, referred to as "allcells." Singlets were then selected using the FSC-A/FSC-W or FSC-A/FSC-H plot. Subsequently, live cells were identified through the LIVE/DEAD Fixable Violet staining (ThermoFisher). Following this, CD45+ cells were gated to isolate immune cells, and subsequent gating was performed based on the expression of surface and intracellular proteins.

- [x] Tick this box to confirm that a figure exemplifying the gating strategy is provided in the Supplementary Information.
